# The Role of the Late Embryogenesis-Abundant (LEA) Protein Family in Development and the Abiotic Stress Response: A Comprehensive Expression Analysis of Potato (*Solanum Tuberosum*)

**DOI:** 10.3390/genes10020148

**Published:** 2019-02-15

**Authors:** Yongkun Chen, Canhui Li, Bo Zhang, Jing Yi, Yu Yang, Chunyan Kong, Chunxia Lei, Ming Gong

**Affiliations:** 1 School of Life Science, Yunnan Normal University, Kunming 650550, China; yongkunchen@hotmail.com (Y.C.); yijingynnu@163.com (J.Y.); yangyu110218@163.com (Y.Y.); yanzi921946483@163.com (C.K.); lcxswx@163.com (C.L.); 2 Joint Academy of Potato Science, Yunnan Normal University, Kunming 650550, China; ch2010201@163.com (C.L.); zhangbo_92@outlook.com (B.Z.)

**Keywords:** late embryogenesis-abundant, abiotic stress, potato, drought, prokaryotic expression

## Abstract

Late embryogenesis-abundant (LEA) proteins are a large and highly diverse family believed to function in normal plant growth and development, and in protecting cells from abiotic stress. This study presents a characterisation of 74 *Solanum tuberosum* LEA (StLEA) proteins belonging to nine groups. *StLEA* genes have few introns (≤2) and are distributed on all chromosomes, occurring as gene clusters on chromosomes 1, 2, and 10. All four *StASR* (*StLEA7* group) genes were concentrated on chromosome 4, suggesting their evolutionary conservation on one chromosome. Expression profiles of *StLEA* genes, in different tissues and in response to hormone and stress treatments, indicated that 71 *StLEA* genes had differential expression levels, of which 68 *StLEA* genes were differentially expressed in response to hormones and stress exposure in the potato. Continuous high expression of *StASR-2*, *StLEA3-3*, *StDHN-3*, *StLEA2-29*, and *StLEA2-14* in different tissues indicated their contribution to plant development processes. *StLEA2-14*, *StLEA2-31*, *StLEA3-3*, *StASR-1*, and *StDHN-1* were upregulated by six abiotic stresses, showing their tolerance to a wide spectrum of environmental stresses. Expression analysis of 17 selected *StLEA* genes in response to drought, salt, heavy metal, heat, and cold treatments by quantitative real-time polymerase chain reaction indicated that StLEA proteins may be involved in distinct signalling pathways. Taken together, *StLEA3*, *StDHN*, and *StASR* subgroup genes may be excellent resources for potato defence against environmental stresses. These results provide valuable information and robust candidate genes for future functional analysis aimed at improving the stress tolerance of the potato.

## 1. Introduction

Late embryogenesis-abundant (LEA) proteins are a type of highly hydrophilic glycine-rich protein with antioxidant, metal ion binding, membrane and protein stabilisation, hydration buffering, and DNA and RNA interaction properties. They play an important role in protecting cells from abiotic stress, and in plant normal growth and development. More importantly, LEA expression is often induced by abiotic stresses such as cold, drought, or high salinity [1,2,3]. LEA proteins are found not only in plant ecosystems ranging from algae to angiosperms, but also in prokaryotes and invertebrates [4,5,6].

In higher plants, many members of the LEA family are present. According to the similarity of amino acid sequences and differences in conserved domains, they can be divided into eight subgroups (LEA1, LEA2, LEA3, LEA4, LEA5, LEA6, dehydrin [DHN], and seed maturation protein [SMP]). Recent studies have shown that abscisic stress ripening (ASR) is also an LEA protein, classified into the LEA7 group [7,8,9]. To date, LEA proteins have been identified in *Oryza sativa* [10], *Hordeum vulgare* [11], *Arabidopsis thaliana* [12], *Prunus mume* [13], *Malus domestica* [14], *Populus trichocarpa* [1], *Solanum lycopersicum* [15], *Pinus tabuliformis* [5], *Dendrobium officinale* [6], and a variety of plants and legumes [4]. The LEA protein was originally discovered during late embryogenesis and later found in roots, stems, and other organs throughout the plant growth phase. They are widely distributed in subcellular compartments, such as the mitochondria, chloroplasts, and cytoplasm. After drought, low temperatures, salt stress, or hormonal treatments, the expression of LEA proteins is upregulated in different development stages and tissues of plants [1,5,16], indicating that plant *LEA* genes play an important role in the response to abiotic stress. Indeed, overexpression of *LEA* genes enhances the abiotic tolerance of transgenic lines, such as the cold tolerance of transgenic *Arabidopsis thaliana*, and the drought- and salt-tolerance of transgenic rice and wheat. However, silencing of the *LEA4* subgroup gene in *A. thaliana* results in sensitivity to water deficit, which may be related to the protective effect of LEA proteins on membrane systems and other biological macromolecules during water deficit [1,12,17,18]. The heterologous expression of *Pinus tabuliformis* LEA (*PtaLEA*) in *Escherichia coli* can also improve cellular salt and heat tolerance [5].

The expression of many LEA proteins is regulated by abscisic acid (ABA), a key hormone in dehydration. During the development of maize embryos, exogenous ABA can upregulate the expression of *LEA4* group members [19,20]. *CuLEA5*, a gene encoding an LEA5 subgroup that plays an important role in fruit ripening in *Citrus unshiu*, is also induced by ABA, cold, and drought stresses [21,22]. In response to environmental changes, ASR adapts to two different conformations: either an α-helix or a polyproline type II (PII) conformation. Low temperature and low pH increase the PII conformation, NaCl increases PII content and disturbs the α-helix conformation, and poly (ethylene glycol) (PEG) and glycerol stabilise the α-helix conformation. This structural plasticity of ASR is critical for plant stress resistance, facilitating their response to drought and interaction with target proteins [9]. DHN improves the freezing tolerance of *E. coli* and plants by increasing the thermal hysteresis value of solution systems to inhibit ice crystallisation [23,24]. These results suggest that each LEA protein may have a unique temporal and spatial role in plant development and the response to abiotic stresses, with obvious functional differentiation seen among subgroups, and among different genes of the same subgroup [5].

The potato (*Solanum tuberosum* L.) is the third largest food crop in the world [25]. Potato production is affected by various environmental stresses, especially because of their shallow roots and poor recovery after water shortages. The potato is very sensitive to water deficit. If drought occurs during its critical growth period, yield and quality will decrease significantly [26,27,28]. Charfeddine et al. [19] identified 29 members of the potato *LEA* family, and the results confirmed the ability of the five *Solanum tuberosum LEA* (*StLEA*) genes of the *DHN* subfamily to respond to salt and drought stress. With the updating of protein domain databases, such as the potato genome, Pfam, and the Conserved Domain Database (CDD), more members of the LEA family have been annotated successively. Due to the obvious functional differentiation among different protein subgroups, functional, evolutionary analyses, and analysis of the distribution of chromosomes of different LEA subgroups, are necessary to study their application to potato stress resistance. In this study, 74 members of the *StLEA* family were identified; their structure, evolutionary relationships, and chromosome locations were analysed, and their expression patterns in different tissues during development and stress tolerance were investigated to deepen understanding of the functions of the *StLEA* family and their applications in potato genetic improvement.

## 2. Materials and Methods

### 2.1. Identification of Late Embryogenesis-Abundant (LEA) Genes in Potato Genomes

Using Pfam ID PF03760 (LEA-1), PF03168 (LEA-2), PF03242 (LEA-3), PF02987 (LEA-4), PF00477 (LEA-5), PF10714 (LEA-6), PF02496 (ASR, LEA-7), PF00257 (DHN), and PF04927 (SMP), the LEA amino acid sequence of the potato was searched and downloaded in the Solanum tuberosum v4.03 database of Phytozome v12.1, and in the Spud DB database (http://solanaceae.plantbiology.msu.edu/) using the keyword “late embryogenesis abundant“. The obtained amino acid sequences were aligned using CD-Search tool (expected value <0.05) in Conserved Domains database (CDD) (https://www.ncbi.nlm.nih.gov/cdd/), and repeated and non-LEA domain sequences were eliminated manually. The physical and chemical properties of StLEA amino acid sequences were analysed using the ProtParam online tool (https://web.expasy.org/protparam/).

### 2.2. Distribution of LEA Genes on Potato Chromosomes

*StLEA* were mapped on potato chromosomes according to the positional information of the *StLEA* genes in the Spud DB database, and displayed using MapInspect software (http://mapinspect.apponic.com/). The segmental duplicated and tandem repeated genes were determined by MEGA X [29]. ClustalW alignment comparisons of all *StLEA* genes with a threshold similarity >75%, and analysis of their genomic locations and tandem duplications, were restricted to a distance range of 100 kb [30].

### 2.3. Structural Characterisation of Potato LEA

Gene structure was obtained through alignment of each *StLEA* gene coding sequence (CDS) to the genomic DNA sequences, and displayed using the Gene Structure Display Server (GSDS) 2.0 online software (http://gsds.cbi.pku.edu.cn/). The Multiple Expectation Maximisation for Motif elicitation (MEME) tool (http://meme-suite.org/index.html) was used to identify conserved domains and motifs of each subgroup of StLEA proteins.

### 2.4. Phylogenetic Analysis of Solanum Tuberosum LEA (StLEA)

Multiple sequence alignments of StLEA proteins were performed using ClustalW ALGN within MEGA X [29]; these were then subjected to phylogenetic tree construction using PHYLOGENY (neighbour-joining method; Poisson correction model; 1000 bootstrap tests).

### 2.5. Expression Profile Analysis of Potato LEA Genes

The RNA sequencing (RNA-Seq) data used for generating gene expression levels were downloaded from the Spud DB database. These data were sequenced from the heterozygous diploid (RH89-039-16 (RH)) and the doubled monoploid potato (Group Phureja clone DM1-3 (DM)). The sequenced tissues included leaves, stems, roots, stolons, young tubers, mature tubers, tuber sprouts, petiole, apices, and flowers, as well as those that received the following treatments: ABA, indole-3-acetic acid (IAA), gibberellin A3 (GA3), 6-benzylaminopurine (BAP), and abiotic stresses such as water stress, mannitol, NaCl, heat (35 °C), primary wounding, and secondary wounding [31]. Gene expression profiling was performed using Origin Lab 2018 (OriginLab Corporation, Northampton, MA, USA). All fragments per kilobase of transcript per million fragments sequenced (FPKM) values were plused with 0.00001. In the 3D heat map, the fold change (FC) of gene differential expression was calculated by log_2_ (FPKM_Treatment_/FPKM_Control_) and displayed according to colour; expression was transformed by square root and displayed according to column height.

### 2.6. Quantitative Real-Time Polymerase Chain Reaction Analysis of StLEA Proteins

Hydroponic potato tissue cultured seedlings (tetraploid variety Cooperation-88) were transplanted to perlite medium. Plants were initially irrigated with Hoagland’s nutrient solution and cultured for 15 days. The medium was leached with one-quarter Hoagland’s solution three times. Then, the seedlings were each treated with 150 mmol L^−1^ NaCl, 5 mmol L^−1^ ZnSO4, and 20% PEG6000 at 4 °C and 35 °C for 24 h. High expression level *LEA* genes, including *StLEA1-3*, *StLEA2-1*, *StLEA2-14*, *StLEA2-17*, *StLEA2-21*, *StLEA2-25*, *StLEA2-31*, *StLEA2-40*, *StLEA3-3*, *StLEA6-1*, *StASR-1*, *StASR-2*, *StASR-3*, *StASR-4*, *StDHN-1*, *StDHN-2,* and *StDHN-3*, were collected for quantitative real-time polymerase chain reaction (qRT-PCR) analysis, and templated by cDNA of the aforementioned root and leaf samples. Three independent biological duplicates were performed in this study. All primer sequences used are listed in Supplementary Table S1. The relative expression levels of *StLEA* genes were analysed using the 2^−ΔCt^ method [32] with the reference gene *StEF1α* [33].

## 3. Results

### 3.1. Genome-Wide Identification and Phylogenetic Analysis of Potato LEA Genes

A total of 74 *StLEA* genes were identified from the potato genome, based on keywords and a Pfam ID search of potato genome databases, identification of *Arabidopsis* LEA amino acid homologous sequence alignment, and a Pfam domain search of the CDD database. These 74 genes were divided into nine groups (groups *LEA1*–*LEA6*, *ASR*, *DHN*, and *SMP*) (Table 1) based on conserved domains and a sequence similarity phylogenetic analysis. The largest group was *StLEA2*, which contained 45 members, while the smallest group was *StLEA6*, with only one member. Groups *StLEA1* and *StDHN* each contained five genes, groups *StLEA3*, *StSMP*, and *StASR* each contained four genes, and groups *StLEA4* and *StLEA5* each contained three genes.

The physical and chemical parameters of most StLEA proteins in the same group were similar according to an analysis using the ProtParam online tool. The 74 *StLEA*-encoded amino acids ranged from 80–501; molecular weights ranged from 8.5 kDa (StDHN-4) to 53.3 kDa (StLEA4-2), with an average of 22.5 kDa. Only seven proteins had molecular weights >30 kDa (StASR-1, StLEA2-9, StLEA2-14, StLEA2-35, StLEA2-40, StLEA4-1, and StLEA4-2). The pI values ranged from 4.47 (StSMP-1) to 10.42 (StLEA2-41), with an average of 8.33. Of all StLEA proteins, 73.0% had a pI > 7.0, with the LEA3 group having the largest pI (9.60) and th SMP group having the smallest pI (5.14). A grand average of hydropathicity (GRAVY) index analysis showed that most of the StLEA proteins were hydrophilic. Of these, 10 proteins with a GRAVY index >0 belonged to group LEA2. The most stable protein was StDNH-1, which had a stability index of 15.36, while LEA2-23 had the highest stability index (118.43).

**Figure 1:**
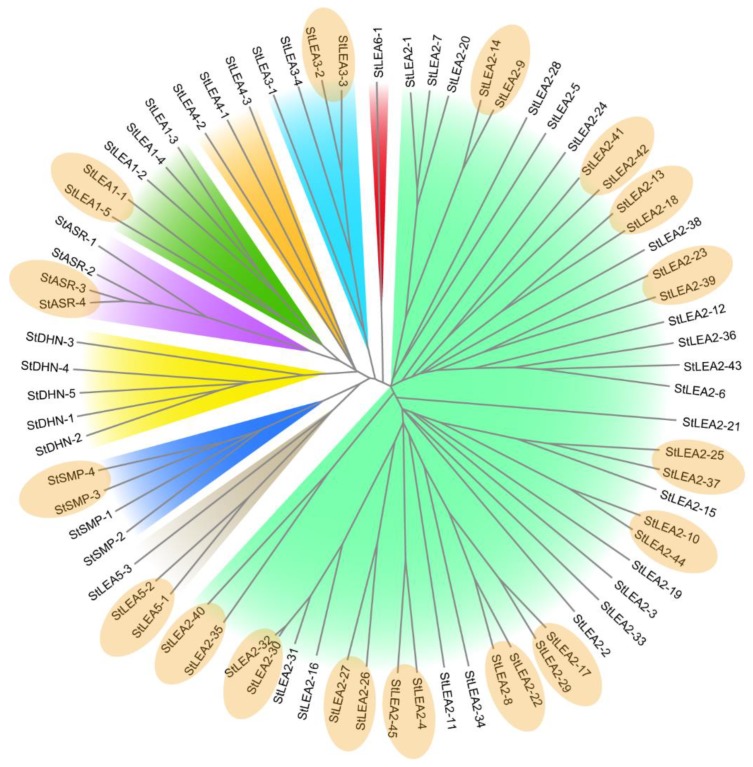
Phylogenetic analysis of potato late embryogenesis-abundant (LEA) proteins. The evolutionary tree of LEA proteins was constructed using MEGA X software [29], with ClustalW alignment, the neighbour-joining method, the bootstrap method, and 1000 repetitions. The LEA1, LEA2, LEA3, LEA4, LEA5, LEA6 ASR, DHN, and seed maturation protein (SMP) subgroups are presented in green, light green, light blue, orange, grey, red, purple, yellow, and blue, respectively. The sister pairs are circled using the same ellipses.

A phylogenetic analysis (Figure 1) showed that groups StLEA2 and StLEA6 were contained in a large branch and were more closely evolutionarily related. The other seven subgroups were contained in another branch and may have a common origin. There were 17 sister gene pairs in the evolutionary tree, with a bootstrap support value >90%. There was one pair in each group for StLEA1, StLEA3, StLEA5, StSMP, and StASR, while there were 12 pairs in 45 members of StLEA2. The high sequence similarity between sister pairs indicated that these genes may have evolved through genome replication events and could have similar functions.

### 3.2. Structural Characterisation of Potato LEA

**Figure 2:**
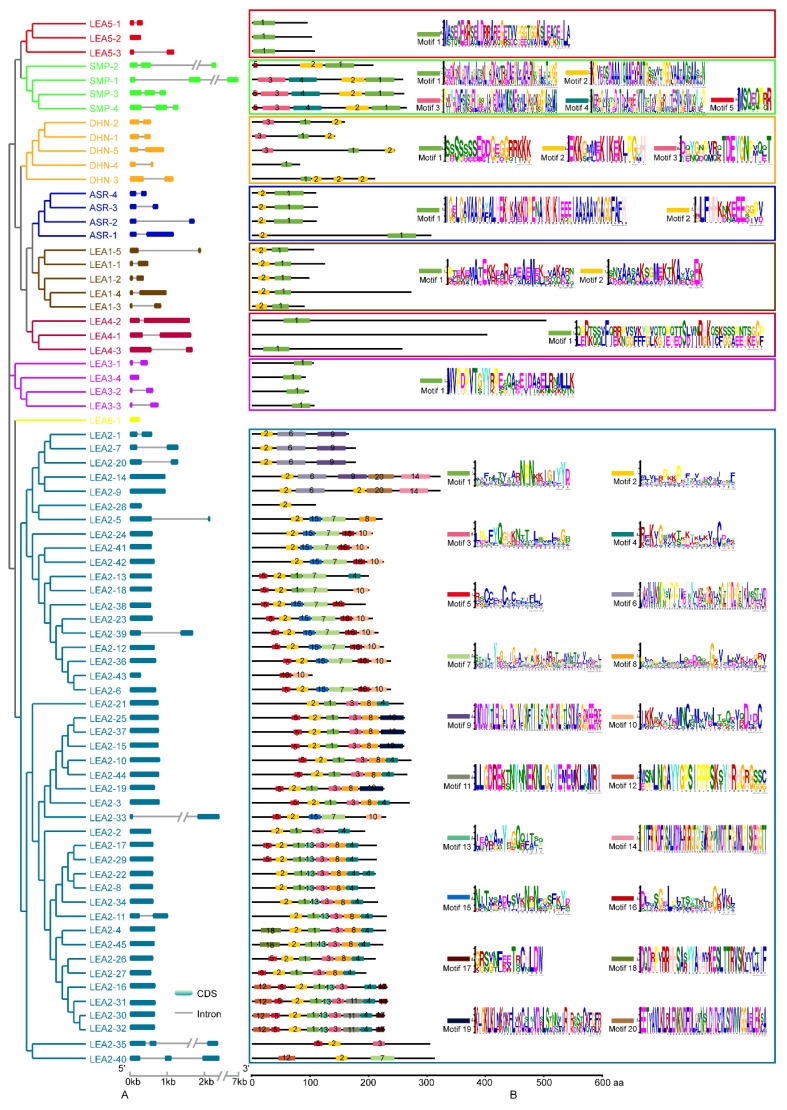
Exon-intron structure and motif distribution of the *StLEA* genes in the potato. (**A**), phylogenetic relationship and exon-intron structure, exon-intron are indicated by wide color bar and gray line, respectively. (**B**), Motif distribution, which was predicted by MEME online tool.

The *StLEA* gene contained few introns, and 39 contained no introns. Only six of the 35 intron-containing LEA proteins contained two introns. The *StLEA6* group contained only one intron-free gene, while subgroups *StLEA1*, *StLEA4*, *StASR*, and *StDHN* contained one intron, which was located in the same clade in the phylogenetic tree (Figure 2A). *SMP* contained two introns, *LEA3* and *LEA5* each had one intron-free gene, and the others were single-intron genes. Among the 45 genes of group *LEA2*, 36 were intron-free, 7 were single-intron genes, and 2 were dual-intron genes (Figure 2A). Two of the seventeen sister gene pairs contained exon-intron gain/loss variations (*StLEA5-1*/*StLEA5-2*, *StLEA2-23*/*StLEA2-39*).

Because of the low similarity of the 74 *StLEA* gene sequences, the MEME online tool was used to analyse the motif structure of each subgroup (Figure 2B). The results showed that except for LEA6, there were conservative motifs specific to each subgroup. Subgroups LEA3, LEA4, and LEA5 each had 1 conserved motif, subgroups LEA1 and ASR had 1 motif, subgroup DHN had 3 motifs, subgroup SMP had 5 motifs, and subgroup LEA2 had 25 motifs. The motifs were conserved in each subgroup. Indeed, all members of LEA2 contained motif 2, all subfamily members of SMP contained motifs 1 and 2, and the DHN subfamily contained the K-segment EKGMMEKIKEKLPGHH, which is rich in lysine residues. These results show that the composition of structural motifs was different among different LEA subgroups, but similar within the same subgroup. Moreover, the motifs encoding LEA domains were relatively conserved, indicating that the functions of StLEA proteins are intergroup specific.

### 3.3. Chromosomal Location and Duplication of Potato LEA Genes

**Figure 3:**
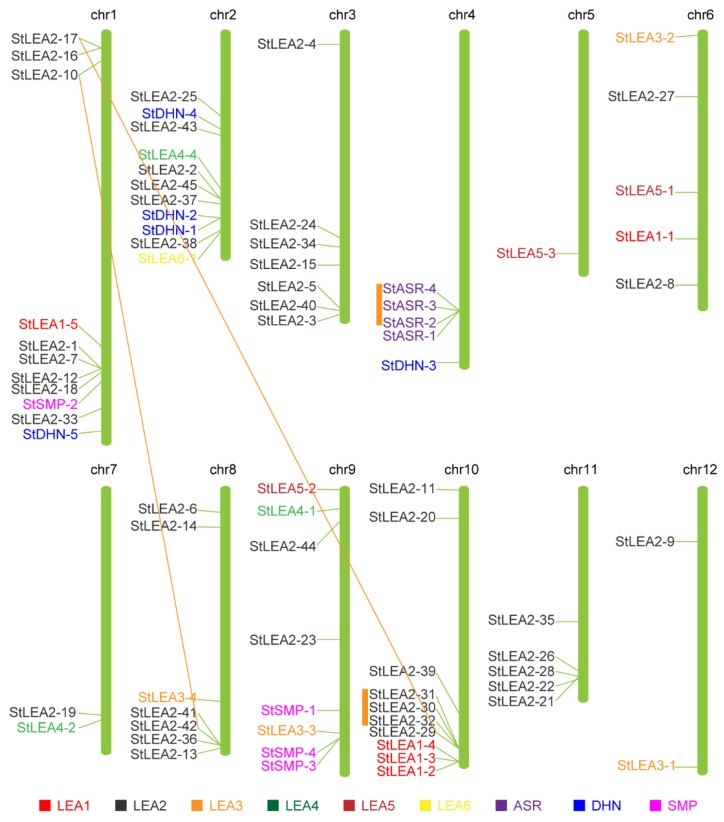
Distribution of *Solanum tuberosum LEA* (*StLEA*) gene family members on potato chromosomes. The genes at two ends of orange lines mean the potential partial duplicated gene pairs. The orange bar indicates the tandem repeated genes. *StLEA1*, *StLEA2*, *StLEA3*, *StLEA4*, *StLEA5*, *StLEA6*, *StASR*, *StDHN* and *StSMP* subgroup are presented in red, black, orange, dark green, dark red, yellow, purple, blue and pink.

Using MapInspect software to analyse genomic position data, 74 *StLEA* genes were distributed on 12 chromosomes, and gene clusters were distributed among specific chromosomal regions (Figure 3); 54 genes were located near the ends of different chromosomes, including chromosomes 1, 2, 3, 4, 8, 9, 10, and 11. According to the chromosomal distribution of the *StLEA* gene, the *StLEA* gene was most densely distributed on chromosomes 1 and 2. Each of these chromosomes contained 11 *StLEA* genes, accounting for 15% of the total number of *StLEA* genes, and 10 *StLEA* genes on chromosome 10. Only one *StLEA* gene was distributed on chromosome 5. The four genes of the *ASR* subgroup were concentrated in a small region of chromosome 4, indicating that they might have a tendency to replicate with conserved evolution within one chromosome. The 45 genes of the *LEA2* subfamily were distributed on 10 of 12 chromosomes to maximise their functions. Among the 17 sister gene pairs, *StASR-3/StASR-4*, *StLEA2-41*/*StLEA2-42*, *StSMP-3*/*StSMP-4*, and *StLEA2-30*/*StLEA2-32* were located on chromosomes 4, 8, 9, and 10, respectively. In accordance with the criteria of Hanada et al. [34], sister gene pairs belong to the same family, are located within 100 kb, and are separated from each other by less than 10 non-homologous genes belonging to tandem duplicates. Although the *StLEA2-17*/*StLEA2-29* sister pair was located on different chromosomes, according to the length of aligned sequence covers more than 80% of the longer gene and the similarity of the aligned region is >70% [35], they had putative segmental duplication events. The *StLEA2-25*/*StLEA2-37* pair was located on the same chromosome but they were far away from each other.

### 3.4. Expression Profile Analysis of Potato LEA Genes in Different Tissues

**Figure 4:**
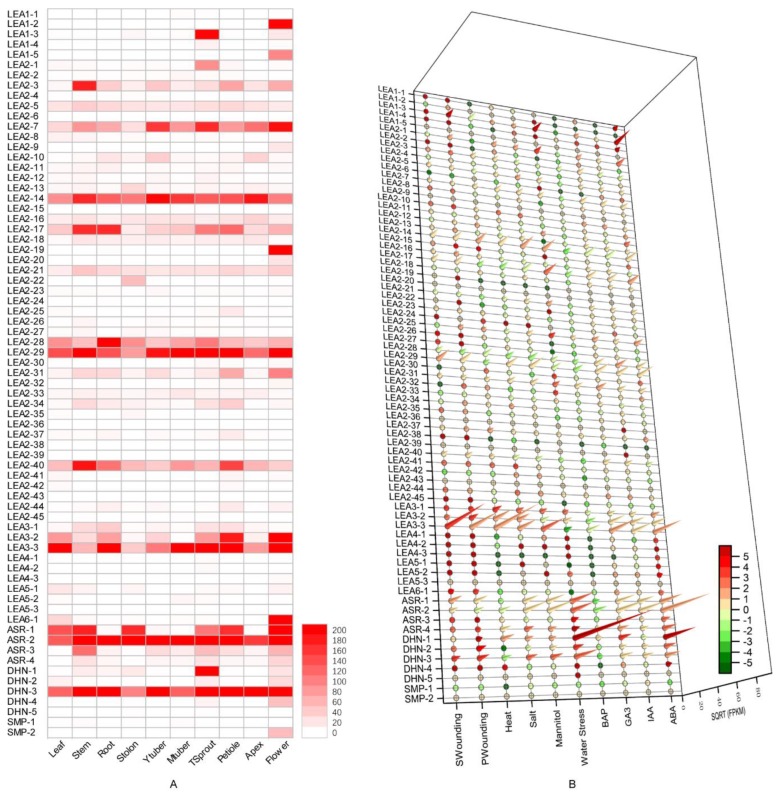
Expression profiling of *StLEA* genes in (**A**) different tissues and (**B**) under different induction conditions.

Apart from *StLEA3-4*, *StSMP-3,* and *StSMP-4*, the expression levels of the remaining 71 *StLEA* genes were significantly different, according to RNA-Seq gene expression data from the Spud DB (Figure 4). Among them, *StASR-2* showed the highest expression level, and there were up to 3864.83 FPKM in stems. Moreover, the FPKM values for roots, tuber sprouts, petioles, shoot apices, and flowers also exceeded 1000, where these values were significantly higher than those of other *StLEA* genes in various tissues. *StLEA 3-3*, *StDHN-3*, *StLEA 2-29*, and *StLEA 2-14* were also highly expressed in various tissues, indicating that they were involved in the normal growth and development of potatoes. In addition, almost half (35) of the *StLEA* genes had a low expression level in each tissue, and the FPKM value was <20. All members of the *StLEA1* and *StSMP* subgroups had lower overall expression levels, except in individual tissues.

The expression levels of *StLEA* genes in different tissues and organs were quite different (Figure 4A). The FPKM variation coefficients of *StLEA* genes in 10 tissues and organs were between 27.4% and 369.7%, excepting the three StLEA proteins without expression data. Some *StLEA* genes were highly expressed in certain tissue types. For example, *StLEA1-2*, *StLEA1-5*, *StLEA2-19*, *StLEA6-1*, *StDNH-4*, and *StSMP-2* were mainly expressed in flowers. There was almost no, or only minimal, expression in other tissues and organs. The FPKM value of *StLEA1-2* reached 197.59 in flowers, but no expression was found in the other nine tissues and organs. *StLEA1-3*, *StLEA2-1*, and *StDHN-1* expression was significantly higher in tuber sprouts than in other tissues. Specifically, the *StDHN-1* FPKM level reached 617.82 in tuber sprouts, which was 28.9- and 36.9-fold higher than the second and third highest expression levels in flowers and stolons, respectively. Some genes had higher expression levels in multiple tissues. The FPKM value of *StASR-1* was >100 in leaves, stems, stolons, tuber sprouts, petioles, and flowers, whereas it was <10 in the other four tissues.

Of the 17 sister gene pairs included in the phylogenetic tree (Figure 1), genes in a given pair typically had different expression patterns (excepting *StSMP-3*/*StSMP-4*, which had no expression data); however, six gene pairs had tandem duplicates or segmental duplications, including *StASR-3*/*StASR-4*, *StLEA2-41*/*StLEA2-42*, *StLEA2-30*/*StLEA2-32*, *StLEA2-17*/*StLEA2-29*, and *StLEA2-25*/*StLEA2-37*, which had similar expression patterns in different tissues. For example, *StLEA2-17* and *StLEA2-29* were expressed in various tissues without any apparent preference.

### 3.5. Effect of Exogenous Hormone Treatment on Potato LEA Expression

Some StLEA proteins can be induced by hormones (Figure 4B). Indeed, 59.5% (44) of the *StLEA* genes were induced by ABA. Fourteen genes were highly upregulated by ABA treatment, with FPKM values >100. Specifically, the FPKM values of *StLEA1-3*, *StASR-2*, and *StDHN-1* were >1000. After ABA treatment, *StLEA1-3* and *StDHN-1* expression was upregulated more than 5-fold, indicating the highest induction level. The induction of *StLEA* by IAA, GA3, and BAP treatment was not as obvious as that by ABA. The expression of 54 *StLEA* genes was significantly inhibited by BAP treatment. *StDHN-1* was highly induced by GA3, and *StLEA1-3* and *StASR-2* were induced not only by ABA, but also by IAA and GA3, demonstrating that many *StLEA* genes are induced by multiple hormones. In addition, ABA, IAA, and GA3 induced the expression of 29 genes, including 1 *StLEA1*, *StLEA4*, *StLEA5*, and *StLEA6* subgroup gene, 17 *StLEA2* subgroup genes, 3 *StLEA3* and *StASR* subgroup genes, and 2 *StDHN* subgroup genes. However, the upregulation and FPKM values in these subgroups were much lower than those of *StLEA1-3* and *StASR-2*. Four members of the *StASR* subgroup were induced by ABA, IAA, and GA3, and inhibited by BAP.

### 3.6. Expression Profiles of Potato LEA Genes under Abiotic Stresses

The expression pattern of *StLEA* genes in response to abiotic stresses, such as drought, high temperature, salt, and mechanical damage were obtained by analysing RNA-seq of data in the Spud DB database (Figure 4B). Apart from six genes, including *StLEA1-5*, *StLEA2-9*, *StLEA2-19*, *StLEA2-20*, *StLEA2-39*, and *StSMP-2*, the other *StLEA* genes were responsive to at least one stress, and the expression patterns were different. The expression levels of the *LEA4*, *LEA5*, *LEA6* and *SMP* subgroups were very low under stress conditions. The expression of *StLEA2-14*, *StLEA2-31*, *StLEA3-3*, *StASR-1*, and *StDHN-1* was induced by six stresses, while the expression of *StLEA2-28* was inhibited by all stresses.

Thirty-four *StLEA* genes were induced by drought. Among these, 11 genes were upregulated by drought stress, including *StLEA1-3*, *StLEA2-1*, *StLEA2-17*, *StLEA2-31*, *StASR-1*–*4*, and *StDHN-1*–*3*. Their FPKM values ranged from 55.04–8636.74, and upregulation (log2FC) ranged from 2.03–12.13-fold. *StDHN-1* expression was upregulated by 12.13-fold and the FPKM value reached 8636.74 after exposure to drought stress. The induction of these genes by mannitol-induced drought stress was at a level similar to that induced by drought stress, but the expression level was significantly lower than that induced by drought stress. Among the 11 drought-induced genes, *StLEA2-17* and *StASR-3* expression was inhibited by mannitol, indicating that mannitol stress could not fully reflect the response of plants to drought. Salt and heat-induced *StLEA* gene expression was significantly lower than that induced by drought, but *StLEA3-1*–*3*, *StDHN-1*, *StDHN-3*, and *StASR-4* expression was significantly induced by salt stress (FPKM 58.37–508.653; log2FC 1.06–2.54). Moreover, the expression of *StLEA3-1*–*3* and *StDHN-1* was also induced by heat. Few studies investigating the induction of *StLEA* by mechanical damage exist. The response of genes to mechanical damage could simulate the response to insect bites. Analysis of *StLEA* expression patterns by primary and secondary wounding treatments showed that mechanical damage induced upregulation of 44 *StLEA* genes, with that of *StLEA3-3* and *StDHN-3* being the most significant. After secondary wounding, the FPKM value of *StLEA3*-*3* was >3000, with a 3.30-fold upregulation (log2FC). The expression and upregulation levels of *StLEA2-14*, *StLEA2-17*, *StLEA2-29*, *StLEA3-2*, and *StASR-1* were also much higher than those of other *StLEA* genes. Based on the above results, the *StLEA3*, *StDHN*, and *StASR* subgroups would be excellent genetic resources to exploit in order to promote stress resistance in the potato.

### 3.7. qRT-PCR Analysis of Potato LEA Genes under Abiotic Stresses

To verify the potential role of *StLEA* genes in abiotic stress, 17 candidate genes of six subgroups with high expression levels were selected, and their expression in potato roots and leaves after treatment with drought, salt, heavy metals, high temperature, and low temperature was analysed by qRT-PCR.

**Figure 5:**
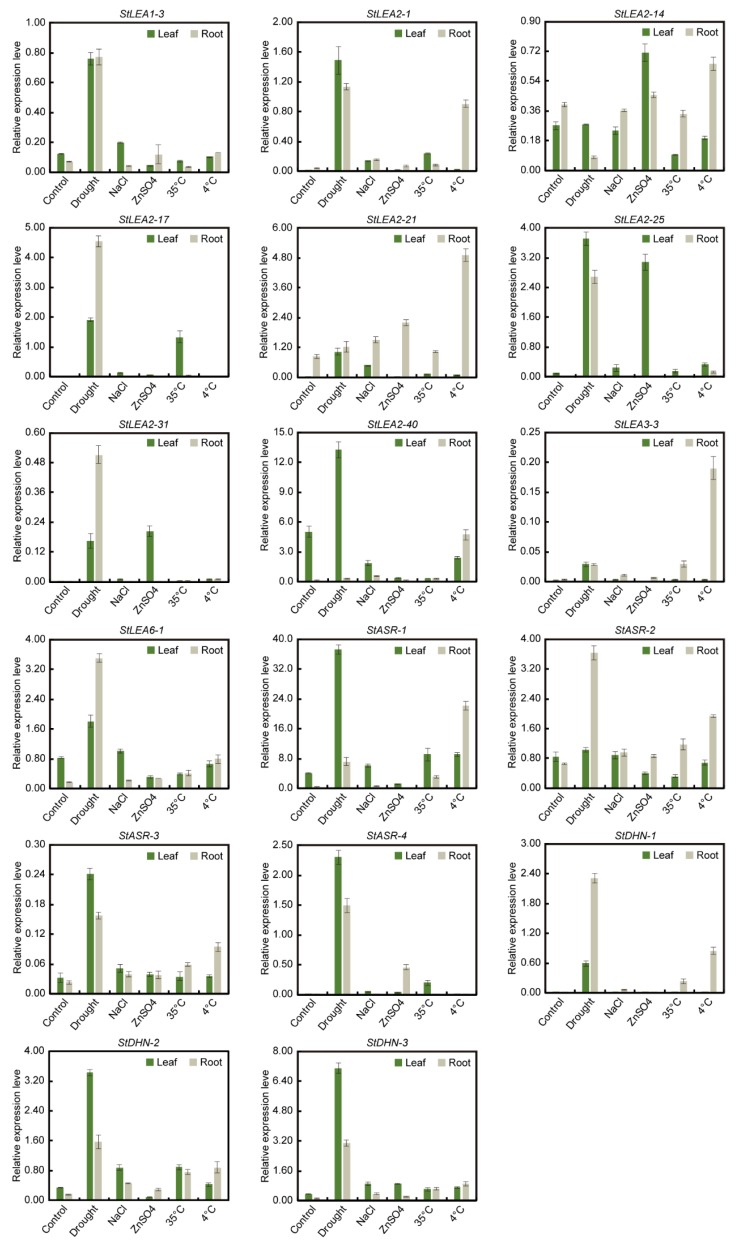
Expression profiles of 17 potato *LEA* genes under various abiotic stresses. Green and light gray green column indicates leaf and root, respectively. Values represent mean ± standard deviation of three replicates.

The results of qRT-PCR showed that the expression of these *StLEA* genes was induced by different stress treatments (Figure 5). Most significantly, drought stress induced the expression of 16 *StLEA* genes in leaves and roots, excepting *StLEA2*-*14*. The highest expression was of *StASR-1* followed by *StLEA2-40*. After drought induction, the expression of five genes (*StLEA2-17*, *StLEA2-31*, *StLEA6-1*, *StDNH-1,* and *StASR-2*) in roots was significantly higher than that in leaves, the expression of three genes (*StLEA1-3*, *StLEA2-21*, and *StLEA3-3*) was similar in roots and leaves, and the other eight genes were more highly expressed in leaves. In response to low temperature, the expression of 15 *StLEA* genes was upregulated, excepting *StLEA2–17* and *StASR-4*. However, compared with the response to drought, 13 of the 15 *LEA* genes were significantly upregulated in roots, especially *StLEA2-1*, *StLEA2-21*, *StLEA3-3*, and *StDNH-1*. The response pattern of LEA to NaCl stress was similar to that to drought stress. Apart from *StLEA2*-*14*, the expression of the remaining 16 *StLEA* genes in leaves and roots was upregulated to varying degrees, but the degree to which they were upregulated was significantly lower than relative to drought-induced expression. The extent to which 17 genes were induced by high temperature was also lower relative to induction by drought. However, the expression of 14 genes, excepting *StLEA1*-*3*, *StLEA2*-*14*, and *StLEA2*-*40,* was also upregulated. The mild upregulation by salt and high temperature stress may be related to insufficient treatment strength. Moreover, the induction of heavy metal stress by *StLEA* was not as obvious as that of the first four stresses. Apart from *StLEA2-14*, *StLEA2-21*, *StLEA2-25*, *StLEA2-31*, and *StASR-4*, the other 12 detected genes were downregulated or minimally different from control levels.

Overall, the expression pattern of most *StLEA* genes under different stresses measured by qRT-PCR analysis was similar to that shown by RNA-seq of data in the Spud DB database. However, some differences existed. For example, under drought stress, the expression of *StLEA2-40*, *StLEA3-3*, and *StLEA6-1* of 17 selected *StLEA* genes in leaves of Cooperation-88 was contrary to that in leaves of RH, which may be related to the plant material and intensity of stress treatment. Most of the *StLEA* genes were induced by drought, low temperature, salt, and high temperature, and a few were induced by heavy metal exposure. The response of *StLEA* genes to abiotic stresses differed between leaves and roots.

## 4. Discussion

### 4.1. Molecular Characteristics and Evolution of the Potato LEA Gene Family

Potato production is threatened by a variety of environmental stresses due to global climate change, especially drought and high temperature, which is driving the need for a greater understanding of the genes in potatoes that are able to cope with these stresses [36]. The *LEA* gene plays an important role in the response to abiotic stress in *O. sativa*, *A. thaliana*, *P. trichocarpa*, and other plants [1,4,10,12]. Charfeddine et al. [19] identified 29 potato *LEA* family members, while more than 50 members of the *LEA* family have been identified in *A. thaliana* and *P. trichocarpa* [1,37], and more than 100 members in *Brassica napus* and upland cotton [3,38]. In this study, 74 genes of the potato *LEA* family were identified by genomic analysis, and were divided into nine subgroups. Their common characteristics include that they have small molecular weights, are rich in hydrophilic amino acids, and contain few introns [3,6]. Indeed, no *StLEA* gene has more than two introns, similar to the trehalose-6-phosphate synthase gene family, which has also been shown to be a stress-response gene family [39]. The small number of introns is a result of genetic evolution, which allows genes to regulate rapidly in response to stress [40].

An analysis of the StLEA protein family revealed that each StLEA group contains conserved motifs that have been identified in other species, including *Oryza, Arabidopsis*, *S. lycopersicum*, and *P. tabuliformis*, [5,10,15,37]. One such motif is a lysine-rich residue K segment contained in the DHN group, indicating that the evolution of the LEA protein was more conserved in plants. However, each group had a uniquely conserved motif. The results of the phylogenetic and expression analyses of StLEA indicated that the function of the LEA protein has group specificity, and members of the same group may have originated from gene expansion within that group [6]. Gene replication plays an important role in the expansion of gene families in a genome. Potato genome sequencing and analysis results have shown that at least two genome replication processes have taken place during the formation of the potato genome [31,41]. Through phylogenetic and chromosomal localisation analysis, 17 sister gene pairs were identified, including four tandem duplicates and two segmental duplications. The *StASR* family was concentrated on chromosome 4, and all of members were clustered in a small region (Figure 3). These results suggest that the chromosomal location of the *StLEA* gene may be the result of gene replication patterns, and the expansion of the gene family may mainly depend on independent duplication of individual sequences, followed by tandem duplication and segmental chromosomal duplication events.

### 4.2. Expression and Function of Potato LEA in Response to Abiotic Stress

Many studies have shown that *LEA* genes play an important role in abiotic stress, especially in drought resistance [38,42]. According to the expression pattern of *StLEA*, some *StLEA* genes (*StASR-2*, *StLEA3-3*, *StDHN-3*, *StLEA2-29*, and *StLEA2-14*) were highly expressed in various tissues (Figure 4A), indicating that they were involved in the normal growth and development of the potato. Other genes have higher expression levels in certain tissues, indicating functional differentiation of genes in the *StLEA* gene family. Moreover, *LEA* subgroups have evolved different adaptive effects against abiotic stresses, as reported in *D. officinale*, *P. tabuliformis*, and upland cotton [3,5,6]. According to the results of an RNA-seq expression analysis, the response of the *StLEA4*, *StLEA5*, *StLEA6*, and *StSMP* subgroups to various stresses was not obvious. The *StLEA3* and *StDHN* subgroups showed a high response to abiotic stress, with only *StLEA1-3* being prominent in the *LEA1* subgroup. The *StLEA2* subgroup was diverse due to its large number of members. Indeed, *StLEA2-14* and *StLEA2-37* were induced by all stresses, while *StLEA2-28* was inhibited by all stresses. Moreover, some genes were upregulated after various stresses, such as *StLEA2-14*, *StLEA3-3*, and *StDHN-1* (Figure 4B), whose functions in potato stress tolerance warrant further study. ASR proteins have been independently reported by Caramelo and Iusem [43]. As their structure is similar to LEA proteins, Hunault and Jaspard [7] classified them into the *LEA* family. It has been reported that the expression of *ASR* is indeed induced by ABA and water stress (Figure 4B), and its expression level is high during fruit ripening [8]. In this study, the expression of all members of the *StASR* group was regulated by ABA, and was also induced by IAA and GA3, but inhibited by BAP. It was additionally induced by various abiotic stresses, especially drought, high temperature, and mechanical damage (Figure 4B). The high expression of *StASR-2* in various tissues indicates that *ASR* genes are also involved in growth and development. These results indicate that the ASR group is a valuable genetic resource in stress resistance research.

The expression patterns of 17 candidate genes in potatoes under drought, salt, high temperature, low temperature, and heavy metal stress were analysed by qRT-PCR. The majority of *StLEA* subgroups were expressed in different tissues in response to these stresses, especially drought and low temperature, followed by salt and high temperature, and a few genes were induced by heavy metal stress (Figure 5). In the tomato, five genes from the *LEA1*, *LEA2*, *LEA4*, and *DHN* groups were upregulated after drought and salt stress [15], while in rice, *LEA1*, *LEA2*, *LEA3* and *DHN* group genes strongly responded to osmotic stress, salt, and ABA exposure [44,45]. In addition, overexpression of *LEA1* also increased drought resistance in rice [45]. Similarly, in *Brassica juncea*, two *DHN* genes were induced by Zn/Cd, and overexpression increased heavy metal resistance in tobacco [46]. These results indicate that the *LEA* genes play important roles in plant resistance to various abiotic stresses. 

In conclusion, a total of 74 *StLEA* genes were identified in *S. tuberosum* and classified into nine groups. Chromosomal location and duplication analysis revealed that 74 *StLEA* genes were distributed in all *S. tuberosum* chromosomes with some gene clustering. All *StLEA* genes contained the LEA motif and had few introns. *StLEA* genes belonging to the same group exhibit similar gene structures. The knowledge garnered from this study may increase understanding of *LEA* genes in *S. tuberosum* in order to lay the foundation for further investigations of the functions of LEA proteins.

## Figures and Tables

**Table 1:** Description of late embryogenesis-abundant (*LEA*) genes identified from the potato genome.

Spud ID	Name	Amino Acid Number	MW	pI	Instability Index	Aliphatic Index	GRAVY
PGSC0003DMG400002093	LEA1-1	123	13,268.01	9.4	48	71.54	−0.715
PGSC0003DMG400011437	LEA1-2	96	10,361.49	9.22	28.3	38.85	−1.161
PGSC0003DMG400011438	LEA1-3	88	9261.33	9.22	17.2	30.34	−1.116
PGSC0003DMG400011439	LEA1-4	271	26,628	8.19	3.02	44.06	−0.589
PGSC0003DMG400001819	LEA1-5	104	11,515.89	5.94	29.76	65.96	−0.828
PGSC0003DMG400000066	LEA2-1	163	17,950.67	4.74	20.54	96.2	−0.153
PGSC0003DMG400002090	LEA2-2	191	22,310.06	9.47	31.39	102.88	0.103
PGSC0003DMG400002586	LEA2-3	267	29,063.08	10.27	36.24	83	−0.136
PGSC0003DMG400005073	LEA2-4	227	25,846.5	9.93	52.17	82.03	−0.292
PGSC0003DMG400005685	LEA2-5	221	25,174.21	9.06	35.71	103.62	−0.015
PGSC0003DMG400005783	LEA2-6	235	26,022.56	9.87	43.17	100.72	−0.039
PGSC0003DMG400000067	LEA2-7	175	19,281.12	4.57	33.54	101.83	−0.189
PGSC0003DMG400005898	LEA2-8	208	23,404.16	9.08	48.26	103.51	0.089
PGSC0003DMG400006460	LEA2-9	320	35,591.61	5.34	26.71	89.47	−0.463
PGSC0003DMG400008711	LEA2-10	270	29,709.53	9.77	45.43	87.63	−0.15
PGSC0003DMG400011296	LEA2-11	228	25,291.92	9.69	50.24	105.88	0.133
PGSC0003DMG400000069	LEA2-12	223	24,906.2	9.68	19.77	94.71	−0.111
PGSC0003DMG400012142	LEA2-13	197	21,631.87	9.1	35.79	85.53	0.197
PGSC0003DMG400013715	LEA2-14	320	35,609.51	4.83	24.23	96.19	−0.41
PGSC0003DMG400015214	LEA2-15	258	29,131.51	10.1	36.09	102.33	−0.092
PGSC0003DMG400016390	LEA2-16	229	26,053.18	8.97	43.15	77.42	−0.154
PGSC0003DMG400016420	LEA2-17	211	23,915.69	9.44	40.96	88.67	−0.132
PGSC0003DMG400000115	LEA2-18	198	21,541.04	9.69	20.03	85	0.171
PGSC0003DMG400018355	LEA2-19	224	25,280.38	8.72	49.75	87.95	−0.169
PGSC0003DMG400019407	LEA2-20	175	19,541.28	4.79	25.24	87.94	−0.362
PGSC0003DMG400019632	LEA2-21	257	28,453.37	10.19	46.62	72.18	−0.325
PGSC0003DMG400019639	LEA2-22	209	23,687.5	9.36	38.63	108.56	0.115
PGSC0003DMG400020863	LEA2-23	204	22,542.59	9.53	34.85	118.43	0.245
PGSC0003DMG400020886	LEA2-24	204	23,102.85	9.7	34.46	87.94	−0.19
PGSC0003DMG400021454	LEA2-25	259	29,397.61	10.06	40.41	92.12	−0.344
PGSC0003DMG400000476	LEA2-26	209	24,030.01	10.05	40.72	98.76	−0.191
PGSC0003DMG400024324	LEA2-27	193	22,002.62	9.83	36.55	103.52	−0.051
PGSC0003DMG400026335	LEA2-28	107	12,026.84	6.58	43.24	93.74	−0.077
PGSC0003DMG400028151	LEA2-29	211	24,106.86	9.59	52.84	91.8	−0.153
PGSC0003DMG400028152	LEA2-30	225	26,077.18	9.29	42.62	86.18	−0.279
PGSC0003DMG400028153	LEA2-31	230	26,121.29	9.11	46.83	89	−0.219
PGSC0003DMG400028235	LEA2-32	225	26,126.21	9.3	43.04	86.62	−0.318
PGSC0003DMG400029777	LEA2-33	227	24,670.78	9.41	28.14	105.15	0.257
PGSC0003DMG400000583	LEA2-34	213	23,996.84	9.41	31.45	90.56	−0.098
PGSC0003DMG400032803	LEA2-35	302	34,188.98	9.11	42.42	86.46	−0.198
PGSC0003DMG400037897	LEA2-36	235	26,782.17	9.59	51.48	95.7	−0.196
PGSC0003DMG401019715	LEA2-37	260	29,590.68	9.57	46.77	91.08	−0.217
PGSC0003DMG401021419	LEA2-38	192	21,050.61	9.53	25.77	100.05	0.177
PGSC0003DMG401027691	LEA2-39	214	24,317.33	9.55	40.92	92.94	−0.204
PGSC0003DMG402002623	LEA2-40	310	34,258.25	9.75	55.92	76.03	−0.324
PGSC0003DMG400012315	LEA2-41	197	22,684.33	10.42	52.16	90.56	−0.23
PGSC0003DMG400012355	LEA2-42	223	25,274	9.68	33.41	87.4	−0.217
PGSC0003DMG400042041	LEA2-43	101	11,316.65	10.02	18.01	107.03	0.242
PGSC0003DMG400002014	LEA2-44	263	29,204.88	9.97	43.83	87	−0.222
PGSC0003DMG400002082	LEA2-45	222	25,335.22	9.51	41.89	91.76	−0.106
PGSC0003DMG400004703	LEA3-1	97	10,887.16	9.2	41.7	57.22	−0.903
PGSC0003DMG400017936	LEA3-2	89	9786.03	9.66	45.73	66.85	−0.473
PGSC0003DMG400031788	LEA3-3	98	10,402.73	9.89	51.67	73.67	−0.351
Sotub08g019440.1.1	LEA3-4	84	9575.08	9.66	49.98	65	−0.554
PGSC0003DMG400002731	LEA4-1	400	43,122.45	5.46	26.56	51	−1.093
PGSC0003DMG400019976	LEA4-2	501	53,302.67	5.32	29.5	57.56	−0.816
PGSC0003DMG400029706	LEA4-3	255	28,437.98	8.84	39.03	47.61	−1.112
PGSC0003DMG400006648	LEA5-1	93	10,011.84	5.91	55.08	40.97	−1.446
PGSC0003DMG400008497	LEA5-2	100	10,855.84	9.1	60.31	38.1	−1.469
PGSC0003DMG400041241	LEA5-3	105	11,740.47	10.07	68.21	68.67	−1.017
PGSC0003DMG400024942	LEA6-1	88	9221.92	5.11	42.02	46.7	−1.125
PGSC0003DMG400003530	DHN-1	140	14,534.01	7.07	34.82	15.36	−1.268
PGSC0003DMG400003531	DHN-2	157	16,659.06	7.23	21.86	36.69	−1.214
PGSC0003DMG400009968	DHN-3	209	23,673.14	5.24	63.91	47.56	−1.499
PGSC0003DMG400015495	DHN-4	80	8544.27	5.9	29.91	37.88	−1.31
PGSC0003DMG400030949	DHN-5	243	25,121.94	7.38	23.94	59.92	−0.715
PGSC0003DMG400019328	SMP-1	257	26,335.96	4.47	40.16	80.54	−0.251
PGSC0003DMG400022470	SMP-2	206	21,737.03	6.78	48.15	74.03	−0.729
Sotub09g023980.1.1	SMP-3	259	26,563.44	4.7	41.15	74.09	−0.353
Sotub09g023990.1.1	SMP-4	263	25,839.64	4.62	35.47	73.57	−0.184
PGSC0003DMG400006661	ASR-1	306	33,956.66	4.92	28.91	19.87	−1.629
PGSC0003DMG400006662	ASR-2	109	12,370.74	6.57	40.22	54.86	−1.294
PGSC0003DMG400006663	ASR-3	111	12,481.95	9.25	41.47	52.97	−1.31
PGSC0003DMG400024093	ASR-4	108	12,158.49	6.65	46.37	52.59	−1.235

Note: MW, Molecular weight (Da); pI, Isoelectric point; GRAVY, Grand average of hydropathicity.

## Supplementary Materials

The following are available online at http://www.mdpi.com/2073-4425/10/2/148/s1, Table S1: Primers for qRT-PCR analysis.
