# Supplementary material for: The Role of the Late Embryogenesis-Abundant (LEA) Protein Family in Development and the Abiotic Stress Response: A Comprehensive Expression Analysis of Potato (Solanum Tuberosum)
Source: Genes (Basel). 2019 Feb 15;10(2):148. doi: 10.3390/genes10020148 (PMC6410179; doi:10.3390/genes10020148)
Supplement: Supplementary file 1 [file genes-10-00148-s001.pdf]

Table S1. Primers for qRT-PCR analysis.

Gene	Forward primer	Reverse primer
qStLEA1-3	GCCAATCTTCAAGAGAAGGGTGAG	GTGGTGGCACCATAACCAGTTCCAG
qStLEA2-1	GCGATGAATTTCGTGTCGGAGAAGG	GTTCCTGATACTATAACCCTGCCGG
qStLEA2-14	GACATGATCAGAGGGAGAGGTAC	GATCTCCAACTTCAATCCTCATCTTC
qStLEA2-17	CCAACACAAACCACTCTCAACAACC	GTTGTGTCTTGGAGGATGAAACGAGG
qStLEA2-21	TCTCTGGCTTATACTCCGTCCTCG	CGTTGTATCCGAAAGCGACACGTC
qStLEA2-25	TCCGTTGGTGCCACGTGGCTCTT	GGTAAACTACGGCTGCTGCGATGC
qStLEA2-31	ACCGTAGTCGCGGTGGTGGTAGTT	AATCGAATTGTGTCAACGTGGCATCAG
qStLEA2-40	GCTGAGATTACTGTCAGGAGTATGG	GCTGACCACTAGCAACAACGATGTC
qStLEA3-3	CCGTTTCTGCTTTACTTAGCAGGCG	GAGCTCAGCAGCGTCAATCTCATTTG
qStLEA6-1	GAAGGATTGCCAGTGGATACAAGTCC	GAAAGCTGAGCTTGAGCAGAGGCAG
qStASR-1	GCTGCTGGTGCCTTTGCTTTGCAT	CCTCAGCTTCCTCCTCTTCTTCCT
qStASR-2	CTGCCGGTGCCTACGCCTTGCAT	AGGTGGTGGTGACCCCCCTCAGCTT
qStASR-3	GCTGCCGGTGCTTACGCCTTGCAT	ATGGTGGTGTCCTCCCTCAGCAG
qStASR-4	GCTGCTGGTGCTTTCGCCTTGCAT	AGTAGTGGTGTCCTCCCTCAGCTT
qStDHN-1	TCGATCCGACAGCTCTAGCTCGTC	TCAATGCATCCCAGGGATCTTGTCC
qStDHN-2	CAGCTCTAGTTCCAGCAGCTCTTC	CCATGATGGCCTGGAAGCTTCTCCT
qStDHN-3	GTAGCAGCTCTAGTAGCTCGAGTG	GTCTTCTTATGTCCTCCACCTGGC
qStEF1α	CTGCACTGTGATTGATGCCCCTGGT	CTTCGGGGTGGTAGCATCCATCTTGT
